# Semi-Quantification of Total *Campylobacter* and *Salmonella* During Egg Incubations Using a Combination of 16S rDNA and Specific Pathogen Primers for qPCR

**DOI:** 10.3389/fmicb.2018.02454

**Published:** 2018-11-02

**Authors:** Michael J. Rothrock, Kristina M. Feye, Sun Ae Kim, Si Hong Park, Aude Locatelli, Kelli L. Hiett, John Gamble, Holly Sellers, Steven C. Ricke

**Affiliations:** ^1^Egg Safety and Quality Research Unit, U.S. National Poultry Research Center, United States Department of Agriculture – Agricultural Research Service, Athens, GA, United States; ^2^Department of Food Science, University of Arkansas, Fayetteville, AR, United States; ^3^Department of Food Science and Engineering, Ewha Womans University, Seoul, South Korea; ^4^Department of Food Science and Technology, Oregon State University, Corvallis, OR, United States; ^5^Poultry Management Safety and Processing Unit, U.S. National Poultry Research Center, United States Department of Agriculture – Agricultural Research Services, Athens, GA, United States; ^6^Department of Biochemistry and Biophysics, Oregon State University, Corvallis, OR, United States; ^7^Department of Veterinary Medicine, Poultry Diagnostic and Research Center, University of Georgia, Athens, GA, United States

**Keywords:** *Campylobacter*, *Salmonella*, poultry, eggs, qPCR, semi-quantification

## Abstract

Rapid molecular techniques that evaluate eggs for the presence of foodborne pathogens is an essential component to poultry food safety monitoring. Interestingly, it is not just table eggs that contribute to outbreaks of foodborne disease. Broiler layer production actively contributes to sustaining of foodborne pathogens within a flock. The surface contamination of production eggs with invasive pathogens such as *Salmonella enterica*, *Campylobacter jejuni*, and *Listeria monocytogenes* during embryogenesis results in gastrointestinal tract (GIT) colonization. Pathogens that secure a niche within the GIT during embryonic development are nearly impossible to eradicate from the food chain. Therefore, current monitoring paradigms are not comprehensive because they fail to capture the presence of invasive pathogens within the embryonic GIT rapidly. By developing tools to recognize the pathogens’ presence in the GIT during embryogenesis, producers are then able to spot evaluate broiler eggs for their potential risk as carriers of foodborne pathogens. In this study a novel qPCR assay was developed to semi-quantify pathogen load relative to total bacterial burden. Eggs sampled from three independent production broiler flocks of different ages were assayed for *S. enterica (invA)*, *C. jejuni (HipO)*, and *L. monocytogenes (HlyA)* against total microbial load (*16s*). The eggs were sampled at 1-day post-set within each flock, 2 weeks post-set, after vaccination (at 2.5 weeks) and 1-day post-hatch. The eggs were washed, and the yolk and embryonic chick GIT were collected. The DNA was extracted and subjected to a qPCR assay. The results confirm a novel technique for pathogen monitoring relative to total bacterial load and a unique method for monitoring the dynamics of foodborne pathogen invasion throughout broiler egg production.

## Introduction

Substantial data indicates that the pathogen load innately carries a certain level of risk; therefore, the absolute presence of any pathogen is substantial enough to remove a carcass from the processing line (Rajan et al., 2017). Broiler eggs are unique reservoirs for foodborne disease, with low levels of foodborne pathogens making the detection and recovery of bacterial cells difficult. The egg contamination occurs in one of two routes—through surface contamination during oviposition and via the invasion of eggs by foodborne pathogens (Cox et al., 2000; European Food Safety Authority [EFSA] and European Centre for Disease Prevention and Control, 2012). Once on the egg, invasive pathogens are capable of entering the embryonic chickens’ GIT (Cox et al., 2002; Heyndrickx et al., 2002). The early exposure of the chicken GIT to foodborne pathogens results in the direct colonization of the pre-immune chick, which makes the eradication of these pathogens from flocks extraordinarily difficult, if not impossible (Claud and Walker, 2001).

Therefore, production layer facilities rely on significant monitoring strategies designed for identifying sources of contamination and contamination events. Efforts directed toward reducing the threat of foodborne pathogens in production layer facilities include the monitoring of eggs via surface swabbing, egg washes, egg carton swabbing, and production hen sampling (United States Department of Agriculture-Animal and Plant Health Inspection Services [USDA-APHIS], 2007). The samples are subsequently assayed via microbiological and molecular analyses to determine pathogen load and prevalence. Unfortunately, these methods fail to detect pathogens from the surface that have successfully invaded the embryonic chicken GIT. Being unable to evaluate the embryonic chick and the yolk for pathogen penetrance fails to truly indicate the risk that individual layer flocks may pose to the contamination risk of hatcheries and broiler facilities.

The data presented herein uses a relative 16s rDNA semi-quantitative qPCR assay to quantitate indigenous *S. enterica*, *L. monocytogenes*, and *C. jejuni* against the total bacterial load of eggs. This study uses this technology to investigate the risk of GIT penetrance associated with three ages of broiler hen flocks: new (25 weeks), mid-life (40 weeks), and old (65 weeks). Egg washes (EW) were compared to yolk and embryonic chick GIT pathogen load. The chicks were sampled 1-week post-set, 2 weeks post-set, after *in ovo* vaccination (at 2.5 weeks), and 1-day post-hatch. Evaluating the penetrance of foodborne pathogens at the post-vaccination time-point in eggs can potentially help identify weak-points in egg hatching that make eggs and developing chicks vulnerable to pathogen invasion. Furthermore, this assay uses rDNA, which provides a relative quantitation of the viable bacterial load against the specific pathogen load. While RNA is an attractive target, field applications can be less user friendly. Therefore, by employing a semi-quantitative, DNA based qPCR assay provides data that comprehensively evaluates broiler eggs for pathogen load and provides a rapid method to track the spread of *Campylobacter* and *Salmonella* throughout egg production.

## Materials and Methods

### Broiler Hatchery

**FIGURE 1:**
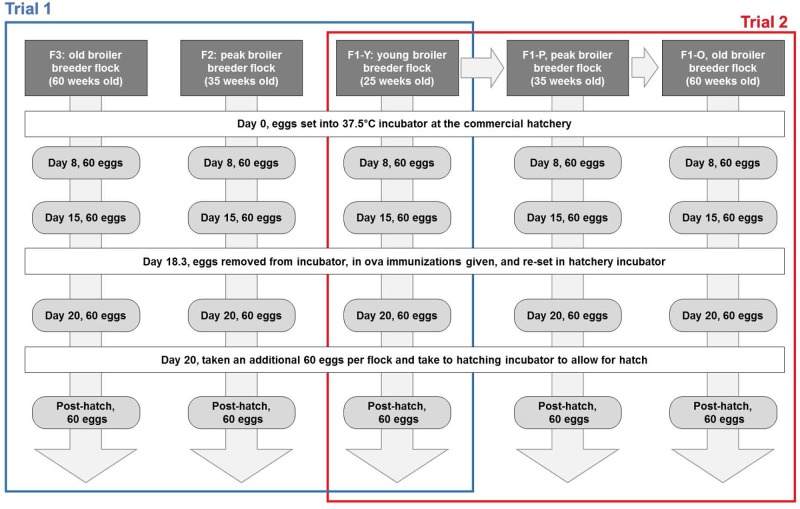
Sampling scheme for this study. Eggs were collected from the commercial hatchery from broiler breeder flocks at three different ages (young, peak, old) for trial 1 (F1, F2, and F3, respectively) and trial 2 (F1-Y, F1-P, and F1-O, respectively). These eggs were collected at four embryonic ages [1 week post set (PS) (T1), 2 weeks PS (T2), 2.5 weeks PS, after *in ovo* immunization (T3), and 1 day post hatch (PH) (T4)]. At every sampling event, triplicate pooled gastrointestinal tract (GIT) and yolk samples were created (*n* = 7 eggs per pool) for embryonic ages. The triplicate pooled egg shell washes (EW) also created for the T3 sampling time only.

A commercial broiler hatchery in the southeastern U.S. provided all of the eggs for this study. Two independent trials were conducted at two different ages of the broiler breeder flocks. Figure 1 shows a visual depiction of the sampling strategy used for this study. In the first trial, eggs from broiler breeder flocks from three separate broiler breeder farms were sampled. The hens were individually caged. These broiler breeder farms were chosen based on the age of the breeders: (1) Young flock (F1) just entering egg production (flock age = 25 weeks), (2) peak egg production flock (F2; flock age = 40 weeks), and (3) older flock producing its last set of eggs prior to culling (F3; flock age = 60 weeks). Throughout the second trial, a single broiler breeder flock (F1-Y; flock F1 from trial 1, representing the young flock age) was followed and was sampled. The F1-Y flock had eggs sampled at the peak (F1-P) and old (F1-O) ages as defined by trial 1. The young and peak flocks from both trials were similarly managed based on integrator’s guidelines. However, the old flocks did experience a proprietary feed formulation change for the old flock at approximately 45 weeks of age. Throughout both studies, the sample collection and processing methods were the same. Once the eggs were set in the commercial hatchery, eggs were collected at four independent time points: (1) 1 week after set (T1: day 8), (2) 2 weeks after set (T2: day 15), (3) after *in ovo* immunization (T3: day 20), and (4) one-day post hatch (T4) (Figure 1).

### Gastrointestinal Tract (GIT) and Egg Yolk Collection

All of the necropsies for this study were performed at the University of Georgia Poultry Diagnostic and Research Center, Athens, GA, United States. All the animal work conducted throughout the study was in accordance with the approved policies and procedures of the University of Georgia Institutional Animal Care and Use Committee (IACUC) number A2010 11-568-Y1-A0. At each sampling event, 60 eggs per flock were collected (F1, F2, F3 or F1-Y, F1-P, F1-O for trials 1 and 2, respectively, Figure 1). Researchers aseptically removed the GIT and yolks from each egg during necropsy. For T1 and T2, eggs from one flock at a time were removed from the 37°C incubator, placed in the Type II biosafety cabinet, sprayed with 0.4% Bioguard (Neogen Corp, Lansing, MI, United States) and allowed to dry prior to sampling. Once the embryos were dry, sterile forceps were used to crack the air cell end of the egg. The embryos were removed from the shell with sterile forceps, the shell was discarded, and the birds were subsequently euthanized by cervical dislocation. The embryos were pooled in groups of seven into a sterile 110 mm^3^ petri dish for sampling. Sterile scissors were used to open the abdominal cavity of the embryos and the intestines were removed with sterile forceps per group. The GIT samples from each group of seven embryos was placed into a small filtered stomacher bag (Seward Laboratory Systems, Inc., Davie, FL, United States). The egg yolks from the eggs of the seven embryos were pooled into large filtered stomacher bags (Seward Laboratory Systems, Inc.) and the remaining amniotic fluid and shell was discarded.

Each of the pooled GIT and yolk samples were weighed and sterile 1x phosphate-buffered saline (PBS) was added to pooled (3:1; 1x PBS volume: GIT mass) to ensure that there was enough homogenate available for all sampling needs. Pooled GIT samples were homogenized via stomaching (Seward Laboratory Systems, Inc.) on maximum speed for 60 s, while the pooled yolk samples were homogenized manually for 60 s.

### Collection of Egg Shell Washes

Egg shell washes (EW) were performed at sampling time T3 to see if the puncturing of the egg shell during *in ovo* immunization introduced egg shell microbial populations into the internal egg environment. Eggs from each breeder flock at T3 were washed in brain heart infusion (BHI) broth by placing each egg, the same ones selected for the selected for embryo harvest in a sampling bag containing 10 mL of BHI broth. The bag was rotated to coat the entire surface of the egg. Then, the eggs were removed, placed on a clean fiberboard flat, placed in the biosafety cabinet and allowed to dry. The eggs were subsequently sprayed with 0.4% Bioguard and allowed to dry. The embryo sampling was conducted as previously described with the following adjustments for the egg washes. For the T4 samples, an extra set of eggs were collected from the commercial hatchery at T3. Those specific eggs were placed into hatching baskets that were arranged by breeder flock, then set into a single stage Natureform Hatcher (NatureForm Hatchery Technologies, Jacksonville, FL, United States), and hatched out at the University of Georgia Hatchery. The chicks were removed from the hatcher by flock, placed in ventilated transport containers and transported to the lab. The chicks were euthanized by group via cervical dislocation, and then placed into sterile 110 mm^3^ petri dishes contained within the biosafety cabinet. The pooled EW samples were centrifuged at 5000 g for 10 min, the supernatant was discarded and the pellet was suspended in 1x PBS. The EW samples were stored at -20 °C until DNA extraction.

### DNA Extraction

Two 0.5 mL aliquots of each pooled sample of either the GIT, yolk, or EW were placed into separate FastPrep Lysing Matrix A tubes (MP Biomedicals, Solon, OH, United States). After lysis, all of the tubes were frozen at -20°C until DNA extraction. The genomic DNA was extracted from the GIT, yolk and EW samples using a hybrid extraction method optimized for poultry samples (Rothrock et al., 2014). Exactly 1 mL of Qiagen ASL buffer (Qiagen, Valencia, CA, United States) was added to each sample tube and vortexed at the maximum setting for 1 min, followed by a more thorough homogenization using the FastPrep 24 (MP Biomedicals) at 6.0 m/s for 45 s. After centrifugation (14,000 × g for 10 min), supernatant was removed, added to a sterile 2 mL tube and incubated at 25°C (yolk) or 95°C in a water bath (GIT, EW) for 5 min. From thereon out, all of the samples were processed using the QIAamp DNA Stool Mini Kit (Qiagen) using the standard stool pathogen detection protocol on the QIAcube robotic workstation (Qiagen). After the automated extraction and purification steps, the two extracted aliquots for each pooled sample were combined in 100 mL sterile molecular grade water using Vacufuge^TM^ Plus (Eppendorf, Hauppage NY, United States), and the DNA concentration in each sample was determined spectrophotometrically using the Take3^®^ plate in conjunction with the Synergy H4 multimode plate reader (BioTek, Winooski, VT, United States).

### qPCR for Bacterial Absolute Enumeration

Total bacteria, *Salmonella* spp. and *C. jejuni*, were targeted using the 16S rDNA (Harms et al., 2003), *invA* (Fey et al., 2004), *hipO* (He et al., 2010), and *hlyA* (Suo et al., 2010) genes, respectively. All DNA extractions analyzed with qPCR were performed on Mastercycler^®^ ep Realplex s2 and s4 thermocycling machines (Eppendorf) in 20 μL reaction mixture was prepared using 10 μL of 2x PerfeCTa qPCR ToughMix, ROX (Quanta BioSciences, Gaithersburg, MD, United States) and 5 μL template of 1:10 diluted sample (containing 10 to 15 ng genomic DNA). Previously published thermocycling protocols were followed and the salient information for all qPCR reactions can be found in Table 1. The PCR amplification efficiency and detection sensitivity were determined by using a series of 10-fold dilutions of standards (10^8^ to 10^1^ copies per reaction) created from purified plasmids for the target gene. The target gene copy number was determined using Mastercycler ep Realplex software (Eppendorf).

**Table 1:** qPCR primer, probes, and protocols used for this study.

Target Group	Gene	Name	Primer/Probe Sequence (5′–3′)	Final concentration (nM)	Tm (°C)	Reference
Total Bacteria	16S	1055F	ATG GCT GTC GTC AGC T	600	58	Harms et al., 2003
		1392R	ACG GGC GGT GTG TAC	600		
		16STaqlll5-BHQ	FAM -CAA CGA GCG –ZEN -CAA CCC – 3IABkFQ	200		
*Salmonella* spp.^1^	*invA*	invA2-F	ATT CTG GTA CTA ATG GTG ATG ATC	400	60	Fey et al., 2004
		invA2-R	GCC AGG CTA TCG CCA ATA AC	400		
*Campylobacter jejuni*	*hipO*	hipO-Cj-F	TCC AAA ATC CTC ACT TGC CAT T	500	60	He et al., 2010
		hipO-Cj-R	TGC ACC AGT GAC TAT GAA TAA CGA	500		
		hipO-Cj-P	FAM- TTG CAA CCT CAC TAG CAA AAT CCA CAGCT-BHQ-1	250		
*Listeria monocytogenes*	*hlyA*	hlyA-LisM-F	ACT GAA GCA AAG GAT GCA TCT G	600	60	Suo et al., 2010
		hlyA-LisM-R	TTT TCG ATT GGC GTC TTA GGA	600		
		hlyA-LisM-P	FAM-CAC CAC CAG CAT CTC CGC CTG C -BHQ-1	200		

*^1^Superscript letter indicates that Salmonella spp. was evaluated using a SYBR kit and not TaqMan.*

### Statistical Analyses

All qPCR data was log_10_-transformed prior to statistical analyses. Two-way ANOVAs evaluated the effect of breeder flock age or embryonic age, as well as their interaction were analyzed via using Prism 6.0 (GraphPad Software Inc., La Jolla, CA, United States). To determine the changes in the pathogen target gene copy numbers relative to the total bacterial population, the log_10_-transformed *invA*, *hipO*, or the *hlyA* values were divided by log_10_-transformmed 16S rDNA values for each pooled samples. The result provided a measure of the relative abundance of each pathogen.

## Results

### Bacterial Population in Trial 1

Time and the flock age dictated pathogen and microbial load. Bacterial loads were low in the GIT samples during the first two weeks post-set for all three broiler breeder flocks (T1 and T2; 4.50–4.54, 4.84–4.52, and 5.61–5.52 log copies/g for F1, F2, and F3, respectively). All three broiler breeder flocks exhibited approximately a 2-log_10_ increase in GIT total bacterial load by 1-day post hatch (T4; 6.07, 7.30, 7.50 log_10_ copies/g for F1, F2, and F3, respectively) (Table 2). The impact of the age of the broiler layer flock was detectable. The youngest flock (F1) exhibited significantly lower GIT total bacterial load as compared to the oldest flock (F3), with the peak age flock (F2) matching the F1 flock early during embryonic development (T1, T2) and the F3 flock later during development (T3, T4). Two of the broiler breeder flocks (F1, F3) yielded low total bacterial loads within the yolk regardless of embryonic age (4.09–4.59 and 4.22–4.58 log_10_ copies/g, respectively), although total bacterial concentrations in the yolks of the F1 and F2 flock both significantly changed by T4 (Table 2). While there were differences in directions of the total bacterial concentration changes (e.g., increase, decrease, no change) between the GIT and yolk samples, two-way ANOVA analyses revealed that breeder flock age, embryonic age, and the interaction of these two ages were considered highly significant for both (Table 3). The age of the broiler breeder flock significantly affected the total bacterial concentrations recovered after T3 for the EW samples (Table 3). The old flock (F3) exhibited greater total bacterial concentrations (6.91 log_10_ copies/mL) compared to the other two flocks (6.56 and 6.63 log_10_ copies/mL for F1 and F2, respectively) (Table 2).

**Table 2:** GIT, Yolk, and EW log transformed qPCR data for three commercial broiler breeder flocks at four times during embryonic development (trial l)^1,2^.

Target		F1				F2				F3			
		T1	T2	T3	T4	Tl	T2	T3	T4	T1	T2	T3	T4
GIT^3^	Total Bacteria (16S)	4.50^B,Y^ (0.10)	4.54^B,Y^ (0.66)	4.25^B,Y^ (0.12)	6.07^A,Y^ (1.33)	4.84,^B,C,Y^ (0.32)	4.52^C,Y^ (0.13)	5.39^B,X^ (0.09)	7.30^A,X^ (0.48)	5.61^B,X^ (0.17)	5.52^B,X^ (0.12)	6.01^B,X^ (0.03)	7.50^A,X^ (0.40)
	*Salmonella (invA)*^6^	0.00^B^ (0.00)	0.00^B^ (0.00)	0.46^A,X^ (0.72)	0.00^B^ (0.00)	0.00 (0.00)	0.00 (0.00)	0.00^Y^ (0.00)	0.00 (0.00)	0.00 (0.00)	0.00 (0.00)	0.00^Y^ (0.00)	0.00 (0.00)
	*C. jejuni (hipO)*	0.48 (1.18)	0.00 (0.00)	0.00 (0.00)	0.49 (1.20)	0.00 (0.00)	0.41 (1.00)	0.67 (1.65)	0.66 (1.61)	0.54 (1.33)	1.38 (1.52)	1.11 (1.73)	0.00 (0.00)
Yolk^3^	Total Bacteria (16S)	4.09^B,Y^ (0.19)	4.30^B^ (0.14)	4.59^A^ (0.06)	4.58^A,Y^ (0.03)	6.99^A,X^ (0.06)	4.40^C^ (0.08)	4.32^C^ (0.61)	5.83^B,X^ (1.92)	4.22^Y^ (0.20)	4.63 (0.29)	4.63 (0.17)	4.56^Y^ (0.08)
	*Salmonella (invA)*^6^	0.00 (0.00)	0.23 (0.57)	0.20 (0.48)	0.30 (0.72)	0.25 (0.61)	0.50 (0.78)	0.00 (0.00)	0.00 (0.00)	0.00 (0.00)	0.26 (0.63)	0.25 (0.61)	0.20 (0.50)
	*C. jejuni (hipO)*	0.61^B,Y^ (0.95)	2.97^A^ (0.15)	0.00^B,Y^ (0.00)	2.89^A,X^ (0.24)	2.88^A,X^ (0.15)	2.85^A^ (0.33)	0.29^B,X,Y^ (0.70)	2.83^A,X^ (0.19)	2.95^A,X^ (0.13)	2.83^A^ (0.13)	0.69^B,X^ (1.07)	0.00^B,Y^ (0.00)
EW^4,5^	Total Bacteria (16S)	ND	ND	6.56^B^ (0.08)	ND	ND	ND	6.63^B^ (0.03)	ND	ND	ND	6.91^A^ (0.14)	ND
	*Salmonella (invA)*^6^	ND	ND	0.00^B^ (0.00)	ND	ND	ND	0.00^B^ (0.00)	ND	ND	ND	1.78^A^ (1.40)	ND
	*C. jejuni (hipO)*	ND	ND	1.56 (1.24)	ND	ND	ND	2.51 (0.23)	ND	ND	ND	1.95 (1.05)	ND

*^1^F1, Young Flock; F2, Peak Flock; F3, Old Flock; T1, 1 week after set; T2, 2 weeks after set; T3, After in ovo immunization (2.5 weeks after set); T4, 1 day after hatch; GIT, Gastrointestinal Tract; EW, Egg Wash.*

*^2^Values represent the Mean (Standard Deviation) of duplicate qPCR runs for triplicate pooled homogenate (GIT, Yolk) or rinse (EW) samples. Samples from 7 eggs constitute a pooled sample. Values represent copies g^-*1*^ (GIT, Yolk) or copes mL^-1^ (EW).*

*^3^Superscript letters A, B, C indicate significant differences (p < 0.05) of qPCR data between embryonic ages (T1, T2, T3, T4) for a given broiler breeder flock (F1, F2, F3). Superscript letters X, Y, Z indicate significant differences (p < 0.05) of qPCR data between broiler breeder flocks (F1, F2, F3) at different embryonic ages (T1, T2, T3, T4).*

*^4^Superscript letters indicate significant differences (p < 0.05) of qPCR data between broiler breeder flocks (F1, F2, F3).*

*^5^ND, not determined.*

*^6^Superscript letter indicates that Salmonella spp. was evaluated using a SYBR kit and not TaqMan.*

**Table 3:** Two-way (GIT, Yolk) and One-way (EW) ANOVA results for three commercial broiler breeder flocks at four times during embryonic development (trial l)^1,2^.

Type		GIT					Yolk					EW				
		SS	DF	MS	F	*P*-value	SS	DF	MS	F	*P* value	SS	DF	MS	F	*P*-value
Total Bacteria (16S)	Breeder Flock Age	20.77	2	10.38	28.73	**<0.0001**	14.11	2	7.05	29.66	**<0.0001**	0.41	2	0.21	22.55	<**0.0001**
	Embryonic Age	51.85	3	17.28	91.76	**<0.0001**	5.93	3	1.98	4.98	**0.0046**					
	Interaction	3.68	6	0.61	3.26	**0.0096**	25.12	6	4.19	10.55	<0.0001					
	Subjects (matching)	5.4	15	0.36	1.92	0.06	3.57	15	0.24	0.60	0.86					
	Residual	8.48	45	0.19			17.86	45	0.40			0.14	15	0.01		
*Salmonella (invA)^3^*	Breeder Flock Age	0.21	2	0.11	2.46	0.12	0.0015	2	0.0008	0.0032	1.00	12.72	2	6.34	9.77	**0.0019**
	Embryonic Age	0.32	3	0.11	2.46	0.08	0.60	3	0.20	0.76	0.52					
	Interaction	0.64	6	0.11	2.46	**0.04**	0.10	6	0.17	0.63	0.71					
	Subjects (matching)	0.65	15	0.04	1.00	0.47	3.51	15	0.23	0.88	0.59					
	Residual	1.95	45	0.04			11.96	45	0.27			9.76	15	0.65		
*C. jejuni (hipO)*	Breeder Flock Age	3.27	2	1.64	1.14	0.35	5.66	2	2.83	11.02	**0.0011**	2.69	2	1.35	1.50	0.26
	Embryonic Age	1.00	3	0.33	0.25	0.86	62.56	3	20.85	91.1	**<0.0001**					
	Interaction	9.00	6	1.50	1.13	0.36	49.90	6	8.32	36.33	**<0.0001**					
	Subjects (matching)	21.49	15	1.43	1.08	0.40	3.85	15	0.26	1.12	0.37					
	Residual	59.55	45	1.32			10.30	45	0.23			13.5	15	0.9		

*^1^GIT, Gastrointestinal Tract; EW, Egg Wash; SS, Sum of Squares; DF, Degrees of Freedom; MS, Mean of Squares.*

*^2^Bolded P-values indicate that ANOVA analyses found those variables to be significant (p ≤ 0.05).*

*^3^Superscript letter indicates that Salmonella spp. was evaluated using a SYBR kit and not TaqMan.*

While the total bacterial load is important, the load of the three main bacterial foodborne pathogens (*Salmonella*, *C. jejuni, L. monocytogenes*) were also targeted by this study. Of these pathogens, only *Salmonella* and *C. jejuni* were detected in the GIT, yolk, and EW samples. *L. monocytogenes* was neither recovered nor quantified in the samples or trials. Across time, in all of the GIT and yolk samples demonstrated that *Salmonella* was only found once in the GIT (F1, T3) (Table 2). *Salmonella* was only detectable in EW from one flock (F3; 1.79 log_10_ copies/mL). Statistically, the age of the broiler breeder flock influences the *Salmonella* and microbial load in the GIT and the EW, but not the yolk (Table 3). *C. jejuni* was the most consistently detected foodborne pathogen in this trial (Table 2). The concentrations of the *C. jejuni* were much higher in many of the yolk samples, with each breeder flock having approximately 2.8 to 2.9-log_10_ copies/g yolk at two or more embryonic ages (T2, T4 for F1; T1, T3, T4 for F2; T1, T2 for F3). Unlike *Salmonella*, there was no significant impact of the embryonic age of the egg nor the age of the production flock alone on *C. jejuni* load. However, a significant interaction occurred between the age of the broiler breeder flock and the egg’s embryonic age on *C. jejuni* load in the yolk (Table 3).

### Bacterial Populations in Trial 2

The effects of breeder flock age on microbial populations within the developing embryo are an important nuance because data gathered can affect flock management. Throughout both studies, temporal effects of age and development were impacted total microbial load. The eggs from a single broiler breeder flock from the first trial (F1) were sampled once that flock reached the peak production (F1-P) and old (F1-O) ages. Low bacterial loads were quantified during the beginning of embryonic develop and were closely followed by approximately a 2-log_10_ increase in total bacterial load by T4 in the GIT samples for the young and old flocks (6.07 and 8.16 log copies/g GIT, respectively) (Table 4). Unlike the first trial, no significant GIT differences were observed in the F1-P flock load. The youngest flock (F1-Y) exhibited the lowest bacterial load; however, the peak flock (F1-P) consistently yielded the highest GIT total bacterial except for one case (T4, F1-O). The total bacterial loads quantified in the yolks were low in the young and old flocks, with the highest microbial load occurring in the peak-age samples (F1-P). The load of microbial populations in the yolk significantly increased between T1 and T4 for both the F1-Y and F1-P flocks. Yet, interestingly, the peak-age yolk concentrations significantly decreased in trial 1 (Table 4). Likewise, the age of the broiler breeding flock and the embryonic age of the egg independently and together significantly affect the total microbial concentrations of the GIT and yolk samples (Table 5). The total microbial load recovered from the surface of the egg (EW) was also significantly impacted by the age of the flock producing the egg, with the F1-Y and F1-P exhibiting greater total bacterial load (6.562 and 6.853 log_10_ copies mL-^1^, respectively) compared to the F1-O flock (5.41 log_10_ copies/mL) (Table 4). This was a complete reversal of what was observed in the first trial.

**Table 4:** GIT, Yolk, and EW log transformed qPCR data at three different flock ages for one commercial broiler breeder flock at four times during embryonic development (trial 2)^1,2^.

Type		F1-Y				F1-P				F1-0			
		T1	T2	T3	T4	T1	T2	T3	T4	T1	T2	T3	T4
GIT^3^	Total Bacteria (16S)	4.42^B,Y^ (0.16)	4.54^B,Y^ (0.66)	4.25^B,Y^ (0.12)	6.07^A,Z^ (1.33)	6.83^X^ (0.25)	6.76^X^ (0.08)	6.74^X^ (0.04)	6.99^Y^ (0.22)	4.51^B,Y^ (0.16)	4.47^B,Y^ (0.09)	4.47^B,Y^ (0.19)	8.16^A,X^ (0.35)
	*Salmonella (invA)^6^*	0.00^Y^ (0.00)	0.00^Y^ (0.00)	0.46^Y^ (0.72)	0.00^Y^ (0.00)	0.00^Y^ (0.00)	0.00^Y^ (0.00)	0.38^Y^ (0.92)	0.00^Y^ (0.00)	3.19^X^ (0.23)	2.60^X^ (1.28)	3.05^X^ (0.25)	3.00^X^ (0.23)
	*C. jejuni (hipO)*	0.47^Z^ (1.15)	0.00^Z^ (0.00)	0.00^Z^ (0.00)	0.49^Y^ (1.20)	5.61^A,X^ (0.71)	5.59^A,X^ (0.19)	5.51^A,X^ (0.02)	4.61^B,X^ (0.04)	4.52^Y^ (0.12)	4.48^Y^ (0.17)	4.37^Y^ (0.27)	4.45^X^ (0.48)
Yolk^3^	Total Bacteria (16S)	4.09^B,Y^ (0.19)	4.30^B,Y^ (0.14)	4.59^A,Y^ (0.06)	4.58^A,Y^ (0.04)	6.47^C,X^ (0.42)	7.23^A,X^ (0.02)	7.26^A,X^ (0.01)	6.84^B,X^ (0.04)	4.18^Y^ (0.20)	4.10^Y^ (0.05)	4.02^Z^ (0.12)	4.04^Z^ (0.14)
	*Salmonella (invA)^6^*	0.00^Y^ (0.00)	0.23^Y^ (0.57)	0.20^Y^ (0.48)	0.30^Y^ (0.72)	0.00^Y^ (0.00)	0.00^Y^ (0.00)	0.28^Y^ (0.69)	0.00^Y^ (0.00)	3.14^X^ (0.21)	2.88^X^ (0.17)	2.95^X^ (0.07)	3.22^X^ (0.13)
	*C. jejuni (hipO)*	0.61^B,Z^ (0.95)	2.97^A,Y^ (0.15)	0.00^B,Z^ (0.00)	2.89^A,Y^ (0.24)	5.60^B,X^ (0.79)	6.70^A,X^ (0.05)	6.75^A,X^ (0.09)	6.36^A,X^ (0.19)	3.05^Y^ (0.24)	3.03^Y^ (0.24)	3.05^Y^ (0.24)	3.13^Y^ (0.08)
EW^4,5^	Total Bacteria (16S)	ND	ND	6.56^B^ (0.08)	ND	ND	ND	6.85^A^ (0.08)	ND	ND	ND	5.41^C^ (0.31)	ND
	*Salmonella (invA)^6^*	ND	ND	0.00^B^ (0.00)	ND	ND	ND	0.00^B^ (0.00)	ND	ND	ND	2.74^A^ (0.21)	ND
	*C. jejuni (hipO)*	ND	ND	1.56^C^ (1.24)	ND	ND	ND	6.23^A^ (0.06)	ND	ND	ND	2.96^B^ (0.35)	ND

*^1^F1-Y, Young Age; F1-P, Peak Age; F1-0, Old Age; T1, 1 week after set; T2, 2 weeks after set; T3, After in ovo immunization (2.5 weeks after set); T4, 1 day after hatch; GIT, Gastrointestinal Tract; EW, Egg Wash.*

*^2^Values represent the Mean (Standard Deviation) of duplicate qPCR runs for triplicate pooled homogenate (GIT, yolk) or rinse (EW) samples. Samples from 7 eggs constitute a pooled sample. Values represent copies g^-1^ (GIT, yolk) or copes mL^-1^ (EW).*

*^3^Superscript letters A, B, C indicate significant differences (p < 0.05) of qPCR data between embryonic ages (T1, T2, T3, T4) for a given broiler breeder flock (F1, F2, F3). Superscript letters X, Y, Z indicate significant differences (p < 0.05) of qPCR data between broiler breeder flocks (F1, F2, F3) at different embryonic ages (T1, T2, T3, T4).*

*^4^Superscript letters indicate significant differences (p < 0.05) of qPCR data between broiler breeder flocks (F1-Y, F1-P, F1-O).*

*^5^ND, not determined.*

*^6^Superscript letter indicates that Salmonella spp. was evaluated using a SYBR kit and not TaqMan.*

**Table 5:** Two-way (GIT, Yolk) and One-way (EW) ANOVA results at three different flock ages for one commercial broiler breeder flock at four times during embryonic development (trial 2)^1,2^.

Type		GIT					Yolk					EW				
		SS	DF	MS	F	*P*-value	SS	DF	MS	F	*P*-value	SS	DF	MS	F	*P*-value
Total Bacteria (16S)	Breeder Flock Age	51.34	2	25.67	91.57	**<0.0001**	118.90	2	59.46	1971.0	**<0.0001**	6.97	2	3.49	98.01	**<0.0001**
	Embryonic Age	46.52	3	15.51	81.72	**<0.0001**	1.41	3	0.47	18.65	**<0.0001**					
	Interaction	27.33	6	4.56	24.01	<**0.0001**	2.20	6	0.37	14.52	**<0.0001**					
	Subjects (matching)	4.21	15	0.28	1.48	0.15	0.45	15	0.03	1.19	0.31					
	Residual	8.54	45	0.19			1.14	45	0.03			0.53	15	0.04		
*Salmonella (invA)^3^*	Breeder Flock Age	130.30	2	65.17	278.20	**<0.0001**	136.90	2	68.44	411.7	<**0.0001**	30.07	2	15.03	1059	**<0.0001**
	Embryonic Age	1.76	3	0.59	2.14	0.11	0.24	3	0.08	0.64	0.60					
	Interaction	1.00	6	0.17	0.61	0.72	0.85	6	0.14	1.11	0.37					
	Subjects (matching)	3.51	15	0.23	0.86	0.61	2.49	15	0.17	1.30	0.24					
	Residual	12.32	45	0.27			5.76	45	0.13			0.21	15	0.014		
*C.jejuni (hipO)*	Breeder Flock Age	355.60	2	177.80	656.20	<0.0001	282.20	2	141.10	12490.00	**<0.0001**	68.89	2	34.44	62.16	**<0.0001**
	Embryonic Age	1.14	3	0.38	1.20	0.32	18.54	3	6.18	37.42	**<0.0001**					
	Interaction	4.48	6	0.75	2.36	**0.05**	29.14	6	4.86	29.41	**<0.0001**					
	Subjects (matching)	4.06	15	0.27	0.85	0.62	1.69	15	0.11	0.68	0.79					
	Residual	14.28	45	0.32			7.43	45	0.17			8.31	15	0.55		

*^1^GIT, Gastrointestinal Tract; EW, Egg Wash; SS, Sum of Squares; DF, Degrees of Freedom; MS, Mean of Squares.*

*^2^Bolded P-values indicate that ANTOVA analyses found those variables to be significant (p ≤ 0).*

*^3^Reflects that the assay was performed using SYBR not TaqMan.*

While *L. monocytogenes* was not detected in any of the samples from this trial, both *Salmonella* and *C. jejuni* were more prevalent in both the GIT and yolk samples from the F1-P (*C. jejuni* only) and F1-O (*Salmonella* and *C. jejuni*) flocks compared to trial 1 (Table 4). *Salmonella* GIT and yolk concentrations were not affected by the embryonic age. Interestingly, the highest GIT, yolk, and EW pathogen loads were found in the oldest broiler breeder flock. Compared to the first trial, the embryonic age affected *C. jejuni* concentrations from both GIT and yolk samples from the F1-P flock. Furthermore, *C. jejuni* significantly decreased by T4 in the GIT samples, whereas they significantly increased in the T4 yolk samples, as compared to the concentrations found at T1. Overall, *C. jejuni* GIT, yolk, and EW concentrations were 1 to 3 log_10_ and 4 to 6 log_10_ higher in the F1-P flock compared to the F1-O and F1-Y (Table 4). Overall, embryonic age was only found to significantly affect *C. jejuni* yolk concentrations, whereas broiler breeder flock age had a highly significant (*p* < 0.0001) effect on *Salmonella* and *C. jejuni* concentrations for all three sample types (Table 5).

## Discussion

Foodborne pathogens that are able to contaminate eggs through vertical integration are a significant concern to the broiler industry. The rise in antibiotic resistant foodborne pathogens is exceptionally well documented, increasing the scrutiny of poultry production from egg to fork (Economou and Gousia, 2015; McCrackin et al., 2015). However, two facts need to be considered concerning eggs and food safety: (1) other foodborne pathogens that cause significant concern, such as *C. jejuni*, are transmitted to the GIT of embryonic broiler chicks, and (2) broiler eggs can be a potential contributor to foodborne outbreaks (Cox et al., 2002; Gole et al., 2014; Mughini-Gras et al., 2014).

Perhaps the most historically common pathogen that reduces production quality and decreases food safety is *Salmonella*. Table eggs are considered to be the other primary source of foodborne *Salmonella* besides poultry meat (Finstad et al., 2012; Howard et al., 2012; Ricke, 2017). *Salmonella*’s ability to infect eggs by invading and colonizing the reproductive tract of hens is a documented route of transmission in poultry with significant consequences to the industry (Gantois et al., 2009; Ricke, 2017). The early establishment of *Salmonella* increases the pervasive threat that the pathogen poses throughout poultry reproduction, rearing, and processing; it can actively disseminate to peripheral organs and contaminate meat. Interestingly, the internal environment of the egg became a more prominent concern after increasing the number of *S. enterica* Enteritidis outbreaks occurred with some of these originating from internal contamination of table shell eggs (St Louis et al., 1988; Henzler, et al., 1994; Cox et al., 2000; Gantois et al., 2009; Howard et al., 2012; Ricke, 2017). In this instance, *S.* Enteritidis was identified by USDA-APHIS from multiple farms and houses on a farm (Henzler et al., 1994). Epidemiologists were able to link *Salmonella* contamination with three independent outbreaks of salmonellosis in the U.S. to those barns, and specifically to the broiler eggs. Environmental sampling of a layer facility is consistently correlated with egg contamination by pathogens (Gole et al., 2014).

Controversy remains as to the ability of other pathogens use their invasion apparatus and colonize the embryonic chicken. Previous studies demonstrate the ability to isolate naturally occurring *C. jejuni* from the circulating blood of broilers as well as the internal organs such as the spleen, liver, adrenal glands, and gall bladder. While this isolation of the pathogen in the periphery can be indicative of the breakdown of stability of the gut barrier, additional evidence has emerged to implicate the vertical transmission of *C. jejuni.* Studies documented the pathogen’s presence in the immature and mature follicles of breeders and the egg shells in commercial laying hens (Cox et al., 2005, 2006, 2007; Richardson et al., 2011; Jones et al., 2016). This is a concern because the establishment of *C. jejuni* in the embryonic gut may have significant consequences for production. The experimental inoculation of chickens by Salmonella and C. jejuni both stimulate the immune and intestinal inflammatory responses (Humphrey et al., 2014; Han et al., 2016). The resulting inflammatory response negatively affects poultry health and production. However, despite the evidence to the contrary and presented herein, the vertical transmission and immune stimulating potential of *Campylobacter* in poultry remains controversial because data challenging the establishment of the vertical route of transmission paradigm continues to emerge (Sahin et al., 2002, 2003; Herman et al., 2003; Newell and Fearnley, 2003; Snelling et al., 2005; Humphrey et al., 2007; Silva et al., 2011).

Current qPCR strategies are also highly correlated to microbial plating techniques to detect total microbial load (Gole et al., 2014) and have been used to quantitate expression of individual genes in *Salmonella* and *C. jejuni* as well as quantitation of both organisms (Gharst et al., 2013; Park et al., 2014; Papić et al., 2017; Neal-McKinney et al., 2018; Ricke et al., 2018). This study directly challenges the sampling paradigm because EW were largely negative throughout the study, with the exception of post-vaccination time point during each trial. Egg washes do not appear to be a good predictor of pathogen invasion within the yolk. Furthermore, this study successfully quantified microbial and pathogen load in an egg. With the exception of *L. monocytogenes*, the pathogens *S. enterica* and *C. jejuni* were identified in the GIT and yolk of the embryonic chick.

The improved detection of pathogens has two goals: (1) improving poultry health which safeguards the food supply; and (2) reducing the transmission of antibiotic resistant pathogens and elements. There is a rise in foodborne pathogens containing antibiotic resistance elements that are capable of disseminating to the naïve microbiota (Nguyen et al., 2016; Kassem et al., 2017; Vita et al., 2018). Therefore, the uncontrolled spread of antibiotic resistant foodborne pathogens increases the risk to the food supply and potentially result in resistant clinical infections (Economou and Gousia, 2015; McCrackin et al., 2015; Battersby et al., 2017; Kassem et al., 2017). Therefore, the detection of these pathogens and the antibiotic resistance profile exhibited is essential to safeguard the food supply. To address these concerns, future studies should include the development of a qPCR system to detect various serovars of *Salmonella* (Mughini-Gras et al., 2014). By expanding on these ideas, the facilitation of the assignment of responsibility and improvement of food safety is innovated.

The implementation of the qPCR strategy delineated in this paper could actively mitigate the risk of the vertical transmission of foodborne pathogens. The sampling of hens occurs monthly or during outbreaks. However, it is well documented that cloacal swabs are not direct indicators of colonization (Langkabel et al., 2014; Battersby et al., 2017). Therefore, by using a “spot check” strategy that looks directly at the egg and the GIT of embryonic chicks is valuable. By doing so, hens with a high rate of transmission of invasive pathogens can be readily identified and culled.

## Author Contributions

MR, AL, KH, JG, SP, and HS designed the experiments, executed the experiments. MR, KF, SK, and SR wrote the manuscript. MR, KF, and SR provided the final analysis, provided ample edits, and finalized the manuscript.

## Conflict of Interest Statement

The authors declare that the research was conducted in the absence of any commercial or financial relationships that could be construed as a potential conflict of interest.
